# ﻿Morphological and molecular analyses reveal two new species of *Termitomyces* (Agaricales, Lyophyllaceae) and morphological variability of *T.intermedius*

**DOI:** 10.3897/mycokeys.95.97156

**Published:** 2023-02-08

**Authors:** Song-Ming Tang, Santhiti Vadthanarat, Jun He, Bhavesh Raghoonundon, Feng-Ming Yu, Samantha C. Karunarathna, Shu-Hong Li, Olivier Raspé

**Affiliations:** 1 Biotechnology and Germplasm Resources Institute, Yunnan Academy of Agricultural Sciences, Kunming 650205, China Mae Fah Luang University Chiang Rai Thailand; 2 School of Science, Mae Fah Luang University, Chiang Rai 57100, Thailand Yunnan Academy of Agricultural Sciences Kunming China; 3 College of Agriculture & Biological Sciences, Dali University, Dali 671003, Yunnan, China Dali University Dali China; 4 Key Laboratory for Plant Diversity and Biogeography of East Asia, Kunming Institute of Botany, Chinese Academy of Sciences, Kunming 650201, China Kunming Institute of Botany, Chinese Academy of Sciences Kunming China; 5 Qujing Normal University, Qujing, Yunnan 655011, China Qujing Normal University Qujing China

**Keywords:** 2 new species, morphology, multi-gene phylogeny, taxonomy, tropical Asia, Yunnan

## Abstract

Two new species, *Termitomycestigrinus* and *T.yunnanensis* are described based on specimens collected from southwestern China. *Termitomycesyunnanensis* is morphologically characterized by a conspicuously venose pileus surface that is grey, olive grey, light grey to greenish grey at center, light grey towards margin, and a cylindrical white stipe. *Termitomycestigrinus* is morphologically characterized by a densely tomentose to tomentose-squamulose pileus showing alternating greyish white and dark grey zones, and a stipe that is bulbous at the base. The two new species are supported by phylogenetic analyses of combined nuclear rDNA internal transcribed spacer ITS1-5.8S-ITS2 rDNA (ITS), the mitochondrial rDNA small subunit (mrSSU) and the nuclear rDNA large subunit (nrLSU). The morphological variability of *T.intermedius*, including five specimens newly collected from Yunnan Province, China, is also discussed. The collections showed variability in colour of the stipe surface and in the shape of cheilocystidia when compared to the original description. Full descriptions of the two new species and of *T.intermedius*, as well as a taxonomic key to the 14 *Termitomyces* species reported from China are provided.

## ﻿Introduction

*Termitomyces* R. Heim (1942a) was established based on the type species *T.striatus* (Beeli) R. Heim (Heim 1942a). *Termitomyces* species are characterized by their obligate symbiotic association with termites (Aanen et al. 2002). Most of species in this genus present a pseudorhiza connected to the termite nests, a usually conspicuous perforatorium (differentiated structure at the centre of the pileus, often in the form of a papilla or umbo), pinkish spore deposit, and smooth broadly ellipsoid to ellipsoid basidiospores (Mossebo et al. 2017; Izhar et al. 2020; Seelan et al. 2020; Usman and Khalid 2020). To date, 59 species of *Termitomyces* have been described worldwide (based on Index Fungorum, accessed on 17 January 2023), of which 14 are reported from China (Wei et al. 2004; Huang et al. 2017; Ye et al. 2019; Tang et al. 2020).

*Termitomyces* species were formerly placed in several different genera, including *Agaricus* (Berkeley 1847), *Armillaria* (Saccardo 1887), *Collybia* (Höhnel and Litschauer 1908), *Schulzeria* (Beeli 1927) and *Lepiota* (Beeli 1927). In 1942, *Termitomyces* was erected with the introduction of seven new species (Heim 1942b). Later, two genera viz. *Podabrella* and *Rajapa*, were segregated from *Termitomyces* by Singer (1945), but these two genera were not broadly accepted as independent (Heim 1977; Gómez 1994; Frøslev et al. 2003; He et al. 2019).

*Termitomyces* species are ecmically important and widely traded as food in the markets of tropical and subtropical areas (Parent and Thoen 1977; Mondal et al. 2004; Chandra et al. 2007; Ye et al. 2019). In India, *Termitomyces* species such as *T.microcarpus* (Berk. & Broome) R. Heim and *T.heimii* Natarajan have also been used for the treatment of diseases such as cold, fever, and fungal infections (Venkatachalapathi and Paulsamy 2016).

Recently, molecular phylogenetic approaches have increasingly been applied to investigate phylogenetic relationships among genera and species of Agaricales (Hofstetter et al. 2002). Through these studies, *Termitomyces* was strongly supported as a genus in Lyophyllaceae, with close relationship to the genera *Calocybe* Kühner, *Tephrocybe* Donk, and *Lyophyllum* P. Karst. (Bellanger et al 2015; He et al. 2019). *Sinotermitomyces* M. Zang, originally described in southwestern China (Zang 1981), was also proven to be a synonym of *Termitomyces* based on the study of type material (Wei et al. 2006).

For the past 70 years, a number of new *Termitomyces* species have been described based only on morphological characteristics. The lack of good illustrations and/or of detailed descriptions made the taxonomy of *Termitomyces* complicated, until the advent of molecular phylogeny. Mossebo et al. (2017) provided molecular markers (nrLSU and mrSSU), bringing more evidence for the classification of *Termitomyces* species. Since then, a series of new *Termitomyces* species have been described from Asia based on combined molecular and morphological data (Ye et al. 2019; Izhar et al. 2020; Seelan et al. 2020; Tang et al. 2020; Usman and Khalid 2020).

During investigations of *Termitomyces* across southwestern China and Thailand, several *Termitomyces* collections were made. Amongst them, two *Termitomyces* species from Yunnan, China, are newly described herein. In addition to the morphological descriptions and illustrations, molecular phylogenetic analyses based on the ITS1-5.8S-ITS2, mrSSU and nrLSU supported the two new species.

## ﻿Materials and methods

### ﻿Studied specimens

Eleven specimens were collected from Southwestern China. Collection locations were subtropical broad-leaved forests in Yunnan Province, where the annual average temperature is 12–22 °C, and the elevation is 1,000–3,500 m (Xiwen and Walker 1986). Three additional specimens were obtained on loan from the Herbarium of Meise Botanic Garden, Belgium (**BR**).

### ﻿Morphological studies

Descriptions of macro-morphological characteristics and habitats were obtained from the photographs and notes. Colour codes were based on Kornerup and Wanscher (1978). Once the macromorphological characteristics were noted, specimens were dried at 40 °C in a food dryer until no more moisture was left, and stored in sealed plastic bags. For microscopy study, dried mushroom materials were sectioned and mounted in 5% KOH solution and 1% Congo red. Microscopic characters such as basidia, basidiospores, and cystidia were observed and photographed using a light microscope (Nikon eclipse 80i) equipped. For microscopic characters’ descriptions, 60–100 basidiospores, 20 basidia, and 10 cystidia were randomly measured, the abbreviations [x/y/z] denote x basidiospores measured from y basidiomata of z collections, (a–) b–c (–d) denote basidiospore dimensions, where the range b–c represents 95% of the measured values while “a”, and “d” are extreme values, L_m_ and W_m_, the average length and width are also given with their standard deviations; Q refers to the length/width ratio of individual basidiospore while Q_m_ refers to the average Q value ± standard deviation. Specimens of the two new *Termitomyces* species were deposited at the herbarium of the Kunming Institute of Botany, Chinese Academy of Sciences (KUN-HKAS) and Mae Fah Luang University herbarium (MFLU).

### ﻿DNA extraction, PCR amplification and sequencing

Genomic DNA was extracted from dry specimens using Ezup Column Fungi Genomic DNA extraction Kit following the manufacturer’s protocol. PCR amplification, PCR product purification, and sequencing. The primers used for nrLSU amplification were LR0R and LR5 (Vilgalys and Hester 1990). The mrSSU region was amplified with *Termitomyces* specific primer pairs viz. SSUFW105 and SSUREV475 (Aanen et al. 2002). The ITS gene region was amplified using the primers ITS1 or ITS5, and ITS4 (White et al. 1990).

### ﻿Sequence alignment and phylogenetic analyses

A total of 29 newly generated sequences and 66 sequences from GenBank were used as ingroup and twelve sequences of *Lyophyllumshimeji* (Kawam.) Hongo, *L.decastes* (Fr.) Singer, *Asterophoralycoperdoides* (Bull.) Ditmar, and *A.parasitica* (Bull.) Singer retrieved from GenBank were used as outgroup (see Table 1). The outgroup taxa were selected based on the phylogeny in Hofstetter et al. (2014). The sequences were aligned with MAFFT version 7 (Katoh and Standley 2013) and checked in Bioedit version 7.0.5 (Hall 2007). The alignment was submitted to Figshare (10.6084/m9.figshare.20472915).

**Table 1.** Names, specimen vouchers, origin, and corresponding GenBank accession numbers of the sequences used in this study. New species are shaded in gray and newly generated sequences are in bold; “*” following a species name indicates that the specimen is the type of that species and “N/A” refers to the unavailability of data.

Taxon	Voucher specimen	Origin	GenBank accession no.			Reference
			ITS	mrSSU	nrLSU	
*Termitomycesacriumbonatus*	LAH36362	Pakistan	MT179687	N/A	MT179690	Usman et al. (2020)
T.acriumbonatus*	LAH35345	Pakistan	MT179688	N/A	MT179689	Usman et al. (2020)
*T.aurantiacus*	DM 152E	Cameroon	N/A	KY809186	KY809234	Willis (2007)
*T.aurantiacus*	tgf 82	Tanzania	N/A	AY127852	AY127804	Mossebo et al. (2017)
*T.brunneopileatus* *	DM392	Cameroon	N/A	KY809225	KY809273	Mossebo et al. (2017)
*T.brunneopileatus*	DM394	Cameroon	N/A	KY809197	KY809244	Mossebo et al. (2017)
*T.bulborhizus*	KM128338	China	N/A	KY809213	KY809261	Mossebo et al. (2017)
*T.floccosus* *	MFLU 19–1312	Thailand	**MT683161**	MN701029	MN633305	Tang et al. (2020)
*T.fragilis* *	HKAS 88912	China	KY214475	N/A	N/A	Ye et al. (2019)
*T.fragilis*	HKAS 88909	China	KY214476	N/A	N/A	Ye et al. (2019)
*T.gilvus* *	BORH/FUMS-A03	Malaysia	N/A	MK478904	MK472701	Seelan et al. (2020)
*T.globulus*	DM770	Cameroon	N/A	KY809204	KY809252	Frøslev et al. (2003)
*T.heimii*	Muid.sn	N/A	N/A	AF357091	AF042586	Moncalvo et al. (2000)
*T.intermedius*	YO198	Japan	AB968241	N/A	N/A	Terashima et al. (2016)
*T.intermedius*	HKAS 117638	China	**ON557369**	**ON557367**	**ON556484**	**This study**
*T.intermedius*	HKAS 117639	China	**ON557370**	**ON557368**	**ON556485**	**This study**
*T.le-testui*	DM150G	Cameroon	N/A	KY809184	KY809231	Mossebo et al. (2017)
*T.le-testui*	KM128346	China	N/A	KY809215	KY809263	Mossebo et al. (2017)
*T.mammiformis*	DM25E	Cameroon	N/A	KY809182	KY809229	Mossebo et al. (2017)
*T.mammiformis*	DM25G	Cameroon	N/A	KY809183	KY809230	Mossebo et al. (2017)
*T.mboudaeinus*	DM223E	Cameroon	N/A	KY809189	KY809237	Mossebo et al. (2017)
T.mediusf.ochraceus	DM602B	Cameroon	N/A	KY809198	KY809246	Mossebo et al. (2017)
*T.radicatus*	MRNo173	Thailand	LC068787	N/A	N/A	Ye et al. (2019)
*T.robustus*	KM142418	Tanzania	N/A	KY809217	KY809265	Mossebo et al. (2017)
*T.robustus*	DM436	Cameroon	N/A	KY809223	KY809271	Mossebo et al. (2017)
*T.sagittiformis*	KM109566	South Africa	N/A	KY809212	KY809260	Mossebo et al. (2017)
*T.schimperi*	DM24E	Cameroon	N/A	KY809181	KY809228	Mossebo et al. (2017)
*T.sheikhupurensis* *	SKP124	Pakistan	MT192217	N/A	MT192228	Izhar et al. (2020)
*T.sheikhupurensis*	SKP224	Pakistan	MT192218	N/A	N/A	Izhar et al. (2020)
*T.singidensis*	tgf74	Tanzania	N/A	AY232687	AY232713	Frøslev et al. (2003)
*T.striatus*	KM142436	Malawi	N/A	KY809219	KY809267	Mossebo et al. (2017)
*T.striatus*	BR5020212704478V	Mali	**OP179298**	**OP179292**	**OP168082**	**This study**
*T.striatus*	BR5020168468769	Rwanda	**OP179297**	**OP179294**	**OP168081**	**This study**
*T.striatus*	BR5020169404421	Congo	**OP179299**	**OP179293**	**OP168080**	**This study**
T.striatusf.bibasidiatus *	DM280B	Cameroon	N/A	KY809193	KY809241	Mossebo et al. (2017)
T.striatusf.subclypeatus *	DM370B	Cameroon	N/A	KY809220	KY809268	Mossebo et al. (2017)
T.striatusf.subclypeatus	DM151C	Cameroon	N/A	KY809194	KY809242	Mossebo et al. (2017)
*T.subumkowaan*	DM260F	Cameroon	N/A	KY809190	KY809239	Mossebo et al. (2017)
*T.subumkowaan* *	DM260B	Cameroon	N/A	KY809227	KY809275	Mossebo et al. (2017)
***T.tigrinus*** *	**HKAS 107560**	**China**	**MT683156**	**MT683152**	**MT679729**	**This study**
***T.tigrinus***	**HKAS 107561**	**China**	**MT683157**	**MT683153**	**MT679730**	**This study**
*T.umkowaan*	HUH-SH5	Pakistan	KJ703245	N/A	N/A	Hussai et al. 2015
*T.upsilocystidiatus* T	MFLU 19–1289	China	**MT683160**	MN636642	MN636637	Tang et al. (2020)
T.yunnanensis*	**HKAS 124501**	**China**	**OP179295**	**OP179290**	**OP168083**	**This study**
***T.yunnanensis***	**HKAS 124502**	**China**	**OP179296**	**OP179291**	**OP168084**	**This study**
**Outgroup**						
*Lyophyllumshimeji*	Lc42	N/A	AF357060	AF357137	AF357078	Hofstetter et al. (2014)
*L.decastes*	JM87/16	N/A	AF357059	AF357136	AF042583	Hofstetter et al. (2014)
*Asterophoralycoperdoides*	CBS170.86	N/A	AF357037	AF357109	AF223190	Hofstetter et al. (2014)
*A.parasitica*	CBS683.82	N/A	AF357038	AF357110	AF223191	Hofstetter et al. (2014)

Phylogenies and node support were first inferred by Maximum Likelihood (ML) from the three single-gene alignments separately, using RAxML-HPC2 version 8.2.12 (Stamatakis 2014) with 1,000 rapid bootstraps, as implemented on the Cipres portal (Miller et al. 2010). Since no supported conflict (bootstrap support value (BS) ≥ 70%) was detected among the topologies, the three single-gene alignments were concatenated using SequenceMatrix (Vaidya et al. 2011). Partitioned Maximum Likelihood (ML) analysis was performed on the concatenated data set, as described above. For Bayesian Inference (BI), the best substitution model for each character set was determined with the program MrModeltest 2.3 (Nylander 2004) on Cipres. The selected models were GTR+I+G for nrLSU, GTR+G for mrSSU, GTR+G for ITS1+ITS2, and K80 for 5.8S. Bayesian analysis was performed using MrBayes version 3.2.7a (Ronquist et al. 2011) as implemented on the Cipres portal (Miller et al. 2010). Two runs of six chains each were conducted by setting generations to 50,000,000 and using the stoprul command with the stopval set to 0.01; trees were sampled every 200 generations. A clade was considered to be strongly supported if showing a BS ≥ 70% and a posterior probability (PP) ≥ 0.90.

## ﻿Results

### ﻿Phylogenetic analyses

The alignments of the nrLSU, mrSSU, 5.8S and ITS1+ITS2 sequences were 538, 354, 157, and 464 characters long after trimming, respectively. The combined data set had an aligned length of 1,516 characters, of which 946 characters were constant, 570 were variable but parsimony-uninformative, and 400 were parsimony-informative.

**Figure 1.**
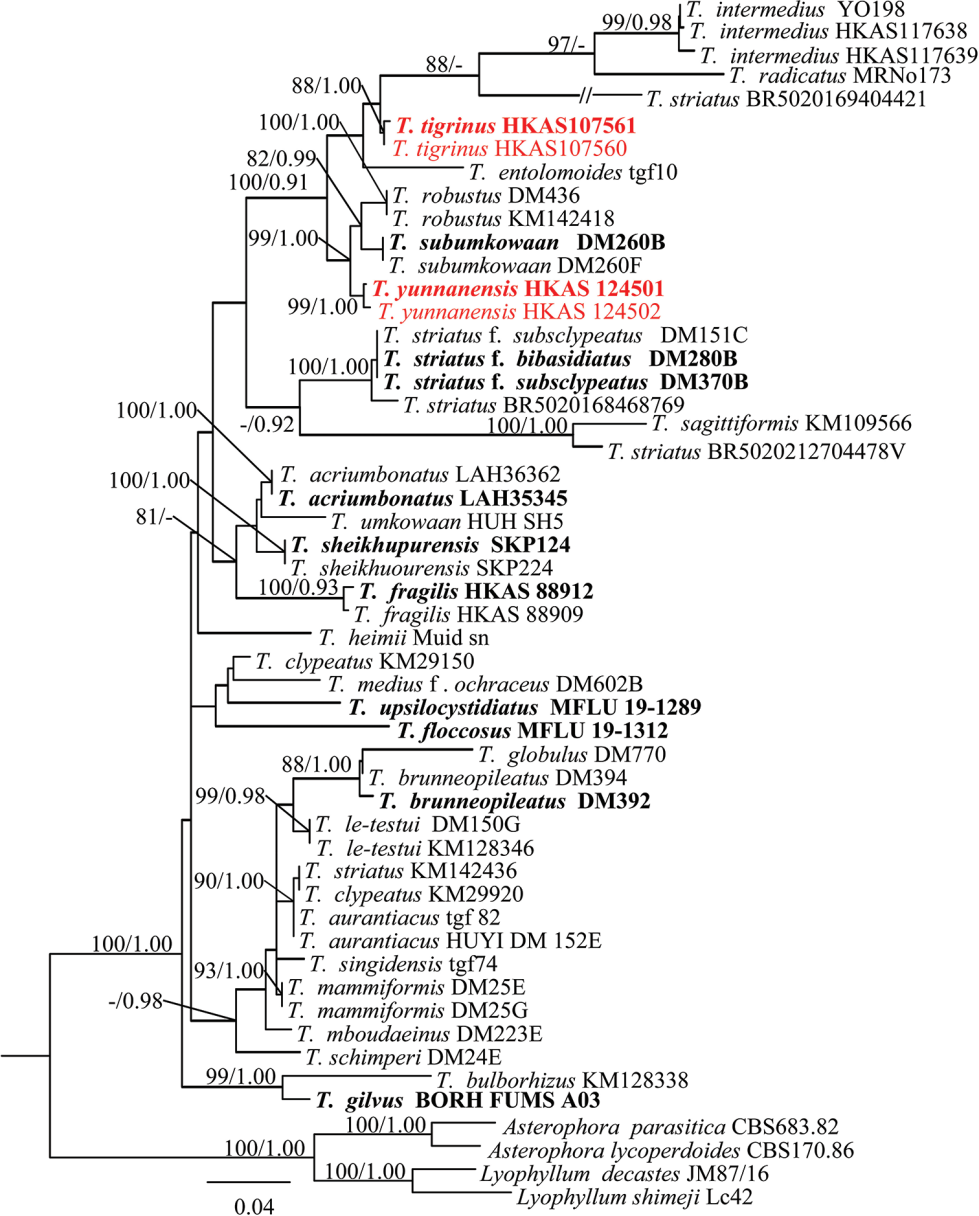
Strict consensus tree illustrating the phylogeny based on the combined nrLSU, mrSSU, 5.8S and ITS1+ITS2 data set. Maximum likelihood bootstrap proportions equal to or higher than 70%, and Bayesian posterior probabilities equal to or higher than 0.90 are indicated at nodes. The two *Asterophora* species and two *Lyophyllum* species were used as the outgroup. The two newly described species are in red. Holotype specimens are in bold.

ML and BI analyses generated nearly identical tree topologies with little variation in statistical support. Thus, only the ML tree is displayed (Fig. 1). Phylogenetic data together with thorough morphological analysis (see below) showed that the two newly described taxa in this study are significantly different from other known *Termitomyces* species.

## ﻿Taxonomy

### ﻿ Termitomyces intermedius

Taxon classificationFungiAgaricalesLyophyllaceae

Har. Takah. & Taneyama

1A279ACB-01E6-545B-A290-5FD1D6326859

Figs 2 , 3

#### Description.

Basidiomata medium-sized. Pileus 4–11 cm in diam., broadly conical or convex when seen from aside, dark grey (1F1), unchanging, often rimose-squamulose in dry condition, squamules easily falling away; margin deflexed to inflexed, undate; perforatorium small and mucronate, dark grey (1F1); context white (1A1), 2–3 mm thick half-way to the margin, tough. Lamellae subventricose, 5–7 mm wide, subfree, crowded, white (1A1) when young, becoming to yellowish white (1A2) when mature; lamellulae in 1–2 tiers; lamellar edge eroded. Stipe central, 3–13 × 1.2–1.6 cm, cylindrical, sometime subbulbous (1.9–2.3 cm) at the base, pale grey (1B1) usually rimose in dry condition, smooth, sometimes irregularly pustulate bumps on the surface; context solid, white, fibrous. Annulus absent. Pseudorhiza terete, tapering downwards; surface pale grey (1B1), smooth; context solid, fibrous. Odour pleasant. Taste not distinctive.

Basidia 43–68 × 10–20 μm, av. 50 ± 8.3 × 14 ± 2.5 μm, clavate, thin-walled, 1-spored or 2-spored, (4-spored basidia not seen); sterigmata 1–2 μm long. Basidiospores [67/9/3] (9.0–) 10.3–14.1 (–14.9) × (5.3–) 5.8–8.9 (–10.2) μm, L_m_ × W_m_ = 11.9 ± 1.1 × 7.3 ± 0.9 μm, Q = 1.4–1.8 (–2.0), Q_m_ = 1.60 ± 0.18, broadly ellipsoid to ellipsoid, colorless, thin-walled, smooth. Hymenophoral trama regular, parallel, 150–230 μm wide, made up of thin-walled, fusiform to narrowly cylindrical hyphae elements 10–23 μm wide, filamentous hyphae abundant, 4–6 μm wide. Subhymenium 8–15 μm thick, with 1–2 layers of ovoid, subglobose, fusiform, ellipsoid or irregular cells, 5–7 × 3–5 μm. Pleurocystidia 40–136 (–169) × 19–34 μm, av. 95 ± 34.1 × 24 ± 9.9 μm, oblong, obovoid or ellipsoid, thin-walled. Lamellar edge heteromorphous, with abundant cheilocystidia. Cheilocystidia 52–114 × 20–29 μm, av. 78 ± 23.3 × 29 ± 8.4 μm, clavate to pyriform, narrowly lageniform, lageniform or broadly lageniform, thin-walled. Pileipellis 2-layered; suprapellis an ixocutis composed of cylindrical hyphae with obtuse apex, thin-walled, hyaline at places in KOH and terminal elements 16–73 × 3–6 μm, av. 46 ± 17.3 × 5 ± 0.9 μm; subpellis made up of inflated elements, 52–131 × 20–27 μm, av. 95 ± 24.8 × 24 ± 8.4 μm. Clamp connections not seen in any tissue.

#### Habitat and distribution.

Basidiomata scattered to gregarious around termite underground nests; occurring in summer. Known from China and Japan.

#### Additional material examined.

**China**. Yunnan Provinces: Kunming city, Shilin county, altitude 1,750 m, 12 July 2019, S.M. Tang 2019071204 (HKAS 117639); Baoshan city, Kejie village, altitude 1,680 m, 3 August 2019, Song-Ming Tang 2019080315 (HKAS 117640); Kejie village, altitude 1,599 m, 3 August 2020, Feng-Ming Yu 2019080321 (HKAS 117641); Dali city, altitude 1,890 m, 21 July 2020, Jun He 202072101 (HKAS 117643); Yuxi city, 1,708 m, 24 July 2020, Jun He 2020072422 (HKAS 117644).

#### Notes.

*Termitomycesintermedius* was originally described from Japan (Terashima et al. 2016), subsequently, it was reported from China in Guangdong province (Huang et al. 2017). Comparison of our specimen (HKAS117638) with *T.intermedius* (TNS-F-48178, Terashima et al. 2016) ITS sequences showed 0.65% difference (4/614 differences, including 3 gaps); nrLSU 100% similarity with GDGM46311 and GDGM46325 (Huang et al. 2017); *tef*1 100% similarity with FB-T1-04 (Kobayashi et al. 2021). Morphologically, our specimen HKAS117638 has narrowly lageniform, lageniform or broadly lageniform cheilocystidia, pileus and stipe surface often rimose-squamulose in dry condition, squamules easily falling away, stipe surface pale grey and sometime subbulbous at the base, while the original description mentioned that *T.intermedius* has broadly clavate to pyriform cheilocystidia and did not mention the pileus and stipe surface in dry condition, stipe white on the surface and cylindrical (Terashima et al. 2016). In *Termitomyces* species, cheilocystidia shape can be variable within the same species. In *T.aurantiacus* (R. Heim) R. Heim for example, cheilocystidia can be rostrate, with median constriction, or moniliform. In *T.mammiformis* R. Heim cheilocystidia can be ovoid, with a median constriction, or narrowly utriform. In *T.schimperi* cheilocystidia can be rostrate, oblong, narrowly utriform, or conical (Heim 1977).

**Figure 2.**
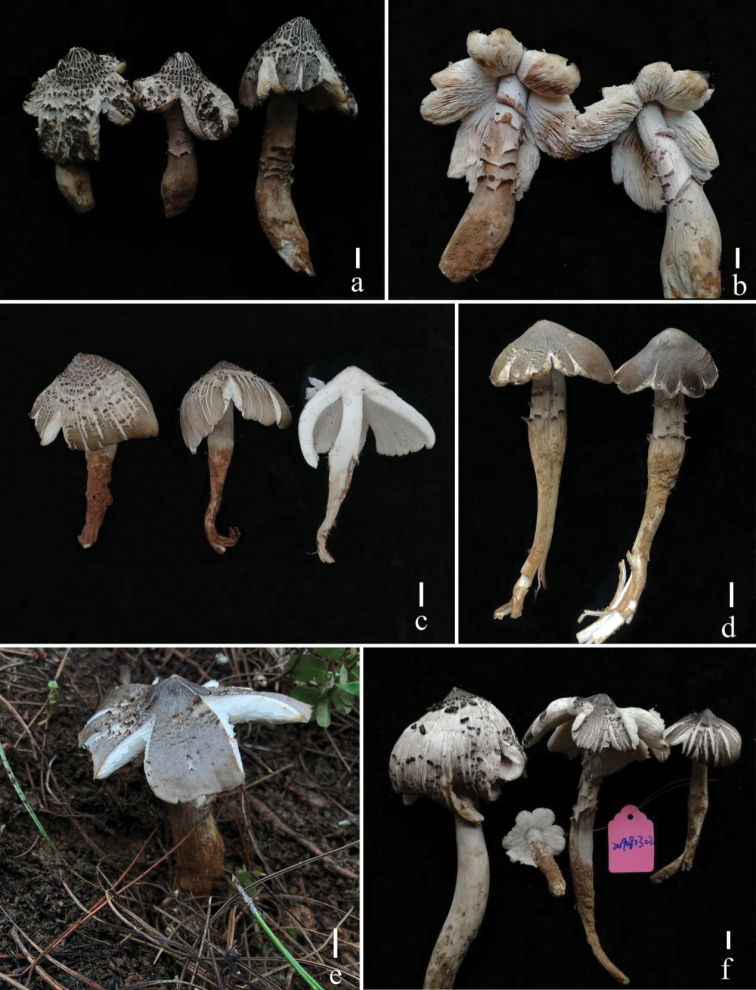
Fresh basidiomata of *Termitomycesintermedius* (**a, b** HKAS 117640, **c** KHAS 117638, **d** HKAS 117644, e-HKAS 117643, f-HKAS 117639). Scale bars: 1 cm. Photographs by Song-Ming Tang.

**Figure 3.**
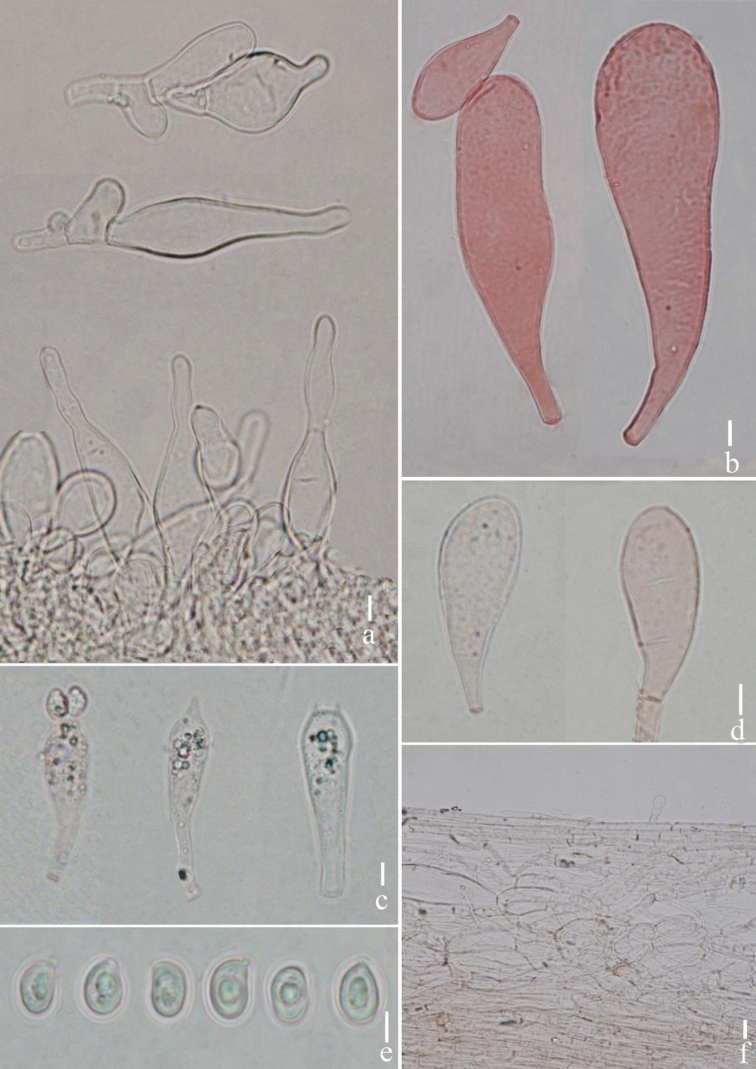
*Termitomycesintermedius***a** cheilocystidia **b** large pleurocystidia **c** basidia **d** small pleurocystidia **e** basidiospores **f** pileipellis. Scale bars: 10 μm (**a–e**); 5 μm (**e**); 20 μm (**f**). Photographs by Song-Ming Tang.

### ﻿ Termitomyces tigrinus

Taxon classificationFungiAgaricalesLyophyllaceae

S.M. Tang & Raspé sp. nov.

2E6B44B8-597F-5D7A-BB7D-7B319222AE0D

836040

Fig. 4

#### Etymology.

The epithet “tigrinus” refers to the alternating greyish white and dark grey zones on the pileus.

#### Type material.

***Holotype*. China**. Yunnan Province: Chuxiong County, Fuming, elev. 1,800 m, 16 July 2019, Song-Ming Tang (***Holotype***: HKAS 107560, ***isotype***: MFLU 22-0143).

#### Diagnosis.

Differs from other *Termitomyces* species in having a regular alternating greyish white and dark grey zones on the pileus, and subclavate stipe.

#### Description.

Basidiomata medium-sized. Pileus 7–9 cm in diam., convex to plano-convex, circular when seen from above, dark grey (1F1–2) at the centre, greyish white (1B1) to grey (1C1–1D1) towards margin, with regular alternating dark grey and greyish white zones, densely tomentose to tomentose-squamulose, hairs grey to drab, in dry conditions, often cracked into large or small scales; margin exceeding lamellae, undate; perforatorium small, as an acute papilla, dark grey (1C1). context 1–2 mm thick half-way to the margin, tough, white (1A1). Lamellae close, ventricose, 3–5 mm wide, adnexed, crowded, white (1A1) at first, then cream to greyish pink when mature; lamellar edge eroded; lamellulae in 1–2 tiers. Stipe 5–7 × 1–3.5 cm, central, subclavate, white (1A1) at the apex, greyish white (1B1) to grey (1C1–1D1) toward the base, smooth; context white (1A1), solid, fibrous. Annulus absent. Pseudorhiza terete, strongly tapering; surface grey (1D1–2) to dark grey (1F1–1F2), smooth; context solid, fibrous. Odour slightly fragrant. Taste not distinctive.

Basidia of two types conspicuously different by the apex of sterigmata being either acute or obtuse, first type rather abundant, clavate, sterigmata apex acute, mostly 4–spored sometimes 1–spored or 2–spored, 25–32 × 7–12 μm, av. 28 ± 2.4 × 11 ± 0.5 μm, sterigmata 1–3 μm long; the second type fewer in number, clavate, sterigmata apex obtuse, mostly 1–spored, 2–spored, sometimes 4–spored, 24–30 × 9–13 μm, av. 26 ± 2.2 × 12 ± 0.7 μm, sterigmata 2–4 μm long. Basidiospores [90/5/2] (6.1–) 7.2–9.6 (–10.1) × (3.3–) 5.2–7.3 (–7.9) μm, L_m_ × W_m_ = 8.1 ± 1.1 × 6.3 ± 0.8 μm, Q = (1.01–) 1.20–2.03 (–2.30), Q_m_ = 1.53 ± 0.20, broadly ellipsoid to ellipsoid, colourless, thin-walled, smooth. Hymenophoral trama regular, element parallel, 51–100 μm wide, made up of thin-walled, ellipsoid to clavate inflated hyphae 16–18 μm wide, filamentous hyphae abundant, 5–10 μm wide. Subhymenium 8–21 μm thick, with 1–2 layers of ovoid, subglobose, fusiform, ellipsoid or irregular cells, 8–11 × 3–6 μm. Pleurocystidia absent. Lamellar edge composed mostly of undifferentiated, basidiole-like cells. Cheilocystidia few, broadly clavate, 17–36 × 9–16 μm, av. 28 ± 2.0 × 14 ± 0.6 μm. Pileipellis 2-layered, suprapellis an ixocutis 22–51 × 5–7 μm av. 30 ± 5.7 × 6 ± 0.5 μm, cylindrical hyphae with obtuse apex, thin-walled, hyaline in KOH; subpellis made up of inflated elements, 31–81 × 7–14 μm, av. 58 ± 7.6 × 10 ± 0.8 μm. Clamp connections not seen in any tissues.

**Figure 4.**
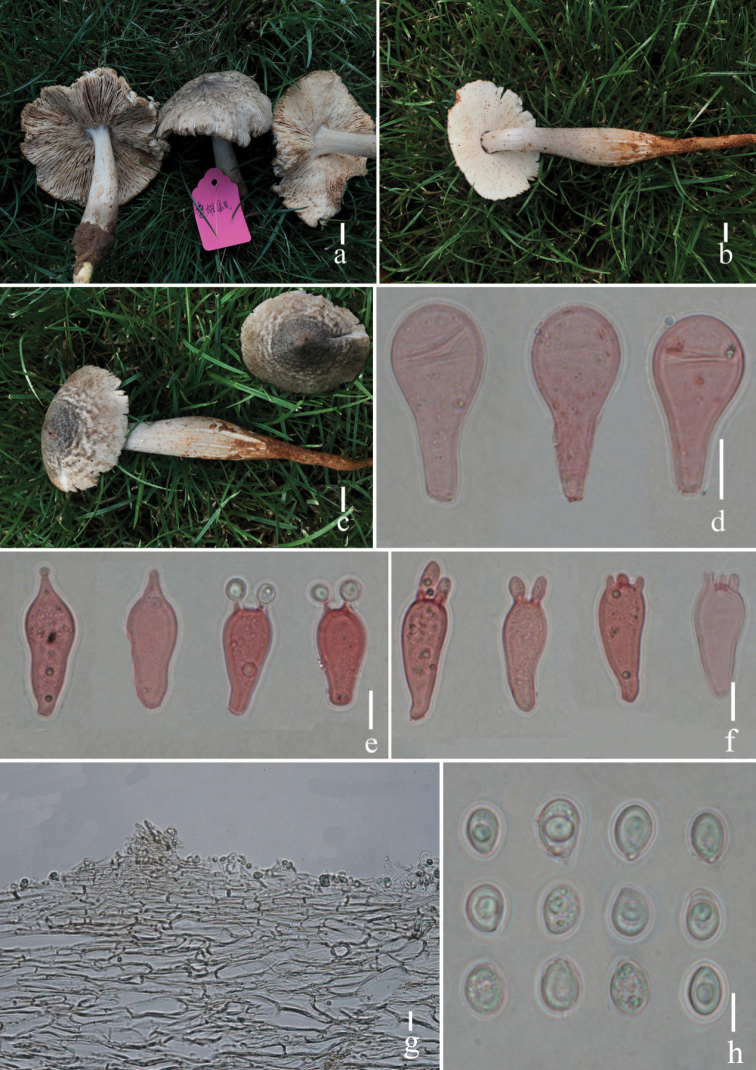
*Termitomycestigrinus***a–c** basidiomata (**a** HKAS 107560, **b, c** HKAS 107561) **d** cheilocystidia **e** basidia with acute sterigmata apex **f** basidia with obtuse sterigmata apex **g** pileipellis **h** basidiospores. Scale bars: 1 cm (**a–c**); 10 μm (**d–f**); 20 μm (**g**); 5 μm (**h**). Photographs by Song-Ming Tang.

#### Habitat and distribution.

Basidiomata scattered on soil with decaying litter under which termites have built their nest. Occurring in summer. So far only known from southwestern China.

#### Additional species examined.

**China**. Yunnan Province, Chuxiong city, 20 July 2019, Jun He (HKAS 107561).

#### Notes.

*Termitomycestigrinus* is distinguished from other *Termitomyces* by densely tomentose to tomentose-squamulose pileus with regularly alternating greyish white and dark grey zones, and a small, dark grey perforatorium as an acute papilla, two conspicuously different types of basidia, broadly ellipsoid to ellipsoid basidiospores, clavate, thin-walled cheilocystidia that are rare (HKAS 107560), or absent (in HKAS 107561).

Morphologically, *T.tigrinus* is similar to *T.robustus* (Beeli) R. Heim in having grey to black stipe. However, *T.robustus* has a blunt perforatorium (Heim 1951), smaller basidia (20–25 × 7–8 μm) and basidiospores (7.0–8.0 × 5.0–5.5 μm) (Otieno 1969).

*Termitomycesentolomoides* R. Heim with *T.tigrinus* in having a tapering upwards stipe, dark grey pileus and greyish white to grey stipe. However, *T.entolomoides* has been originally described from Congo by Heim (1951), has a small basidioma (pileus 3–4 cm diam.), a grey pseudorhiza and relatively smaller basidiospores (6.2–6.6 × 4–4.2 μm; Heim 1951).

In our multi-locus phylogeny, *T.tigrinus* is closely related to *T.intermedius*, *T.radicatus* Natarajan and *T.striatus*. However, *T.intermedius* has a cylindrical stipe, and cheilocystidia clavate to pyriform, narrowly lageniform, lageniform or broadly lageniform (this study). *Termitomycesradicatus* has a pale orange pileus, dark brown perforatorium, relatively smaller pileus (1.5–3.5 cm), and cylindrical stipe (Pegler and Vanhaecke 1994). *Termitomycesstriatus* has a white to ocher pileus, irregularity fibrous striate on the stipe surface, long pseudorhiza (30–100 cm), with squama of various sizes and shapes on the surface cheilocystidia and pleurocystidia pyriform,broadly clavate, cylindrical or ovoid, (20–45 × 11–22 μm; Heim 1977).

### ﻿ Termitomyces yunnanensis

Taxon classificationFungiAgaricalesLyophyllaceae

S.M. Tang & Raspé sp. nov.

23218C71-FA38-5E82-A5E9-4EAF2DF544A8

845183

Figs 5 , 6

#### Etymology.

The epithet “yunnanensis” refers to the holotype coming from Yunnan province.

#### Type material.

***Holotype*: China.** Yunnan province: Kunming city, Shilin county, 20 August 2020, elev. 1580 m, S.M. Tang (***Holotype***: HKAS124501, ***isotype***MFLU 22-0144).

#### Diagnosis.

Differs from other *Termitomyces* species in having a clearly conspicuously venose pileus surface, and an umbonate perforatorium.

#### Description.

Basidiomata medium-sized. Pileus 4–8 cm in diam., at first convex becoming convexo-applanate to plano-concave or concave, medium grey (1E1), olive grey (1E2), light grey (1D1) to greenish grey (1D2) at center, light grey (1D1) towards margin, conspicuously venose surface; margin inflexed when young, becoming straight or reflexed when mature; perforatorium an umbo, ca. 7–9 mm, dark grey (1F1); context 2–4 mm thick half-way to the margin, tough, white (1A1). Lamellae subventricose, free to adnexed, crowded; lamellulae in 1–2 tiers, white (1A1), 3–5 mm wide; lamellar edge eroded. Stipe 3–4 × 1–2 cm, central, cylindrical, rarely subbulbous at the base, smooth; context solid, fibrous, white (1A1). Annulus absent. Pseudorhiza terete, tapering downwards, surface grey (1D1–2) to dark grey (1F1–1F2), smooth; context solid, fibrous.. Odour slightly fragrant. Taste not distinctive.

Basidia of two conspicuously different types by the sterigmata apex acute or obtuse, first type rather abundant, sterigmata apex acute, clavate, mostly 2–spored, sometimes 4–spored, 20–30 × 7–15 μm, av. 25 ± 2.4 × 11 ± 1.8 μm, sterigmata 1–4 μm long; the second type fewer in number, sterigmata obtuse, clavate, mostly 2–spored, sometimes 4–spored, 24–32 × 8–15 μm, av. 27 ± 2.2 × 10 ± 1.1 μm, sterigmata 2–3 (–5) μm long. Basidiospores [139/2/2] 6.5–10.2 (–11.1) × (3.9–) 4.5–8.2 (–9.1) μm, L_m_ × W_m_ = 8.6 ± 1.0 × 5.9 ± 0.8 μm, Q = 1.2–1.8, Q_m_ = 1.47 ± 0.16, broadly ellipsoid to ellipsoid, colorless, thin-walled, smooth. Hymenophoral trama regular, parallel, 150–200 μm wide, made up of thin-walled, ellipsoid to clavate inflated cells hyphae 20–28 μm wide, filamentous hyphae abundant, 3–6 μm wide. Subhymenium 10–20 μm thick, with 1–2 layers of ovoid, subglobose, fusiform, ellipsoid or irregular cells, 7–13 × 3–6 μm. Cheilocystidia 14–37 × 13–23 μm, av. 23 ± 9.1 × 18 ± 4.9 μm, ellipsoid, obovoid to broadly clavate, thin-walled. Pleurocystidia similar to cheilocystidia in shape, 33–50 × 19–32 μm, av. 37 ± 9.1 × 25 ± 5.8 μm. Lamellar edge heteromorphous, more in number of cheilocystidia. Pileipellis 2-layered, suprapellis an ixocutis, 9–39 × 3–5 μm av. 23 ± 8.1 × 4 ± 0.5 μm, cylindrical hyphae with obtuse apex, thin-walled, hyaline at places in KOH; subpellis made up of inflated elements, subcylindrical, 17–49 × 10–18 μm av. 34 ± 9.2 × 13 ± 2.4 μm. Clamp connections not seen in any tissues.

#### Habitat and distribution.

Solitary above underground termite nests; basidiomata occurring in summer. Known from southwestern China.

#### Additional material examined.

**China**. Yunnan Province: Kunming city, Shilin county, Banqiao town, 11 July 2019 alt. 1500 m, J. He (HKAS 124502); ibid, 11 July 2019, alt. 1350 m, S.M. Tang (KHAS 124503); Yuxi city, Eshan county, 7 August 2019, alt. 1480 m, S.M. Tang (HKAS 124517).

#### Notes.

*Termitomycesyunnanensis* is distinguished from other *Termitomyces* species by its clearly striated pileus surface, medium grey, olive grey, light grey to greenish grey at center, light grey towards margin on the pileus surface; perforatorium dark grey and umbonate, thin-walled or thick-walled basidia, ellipsoid, obovoid to broadly clavate cheilocystidia and pleurocystidia.

According to our multi-locus phylogenetic analyses, *T.yunnanensis* was clustered together with *T.subumkowaan* Mossebo and *T.robustus*. However, *T.subumkowaan* has yellowish to brownish grey pileus, obtuse perforatorium concolorous with pileus, stipe cylindrical, bulbous at the base, and pleurocystidia extremely rare (Mossebo et al. 2002; Mossebo et al. 2017). *Termitomycesrobustus* has bigger pileus (7–11 cm), pileus grey, often rimose-squamulose when dry, perforatorium concolorous with pileus, acute and bigger perforatorium (Sitotaw et al. 2015).

Morphologically, *T.medius* R. Heim & Grassé, *T.mammiformis*, *T.griseiumbo* and *T.striatus* are similar to *T.yunnanensis* in having a clearly striated pileus surface. However, *T.medius* has smaller pileus (2.2–2.9 cm), and acute perforatorium, reflexed pileus margin when mature, smaller basidiospores (6–8 × 4–4.8 μm) and basidia (17–20 × 7–7.5 μm), pleurocystidia (25–40 × 12–25 μm) narrowly utriform, ovoid to obovoid (Heim 1977). *Termitomycesmammiformis* has subconical scales on the pileus surface, and an annulus on the stipe (Heim 1977). *Termitomycesgrisumbo* has ochraceous pileus, and relatively bigger pileus (12–15 cm), and smaller basidiospores (5.5–7 × 3.5–4.5 μm), pleurocystidia abundant and polymorphic, clavate to pyriform, with one or more transverse septa (Mossebo et al. 2002).

*Termitomycesstriatus* originally described from Sierra Leone (Africa), has clear striae on the pileus, ring of scales on the pseudorhiza, and small basidiospores (6.5–7.7 × 4–5 μm) (Heim 1977). However, *T.striatus* was divided 10 formae (Mossebo et al. 2009), namely f. annulatus, f. striatus, f. ochraceus, f. bibasidiatus, f. griseus, f. griseiumboides, f. subumbonatus, f. brunneus, f. pileatus and f. subclypeatus. However, according to the phylogenetic analysis of nrLSU and mtSSU sequence in Mossebo et al. (2017), f. striatus (tgf99), f. bibasidiatus (DM280), f. subumbonatus (DM208) and f. subclypeatus (DM151, DM370) were in a different species-level clades, and should therefore be considered as different species. Termitomycesf.bibasidiatus , f. subumbonatus , f. subclypeatus were originally described from Cameroon (Africa) and these species are morphologically different from *T.yunnanensis. Termitomyces* f. bibasidiatus has relatively long pseudorhiza (20–60 cm), pale, reddish grey to brownish orange yellow pileus, and globose to ovoid pileipellis cells (Mossebo et al. 2017). An annulus is present in Termitomycesf.subumbonatus (Mossebo et al. 2002), but absent in *T.yunnanensis*. Termitomycesf.subclypeatus has whitish orange to pale orange pileus with a greyish yellow to brownish orange perforatorium (Mossebo et al. 2017).

**Figure 5.**
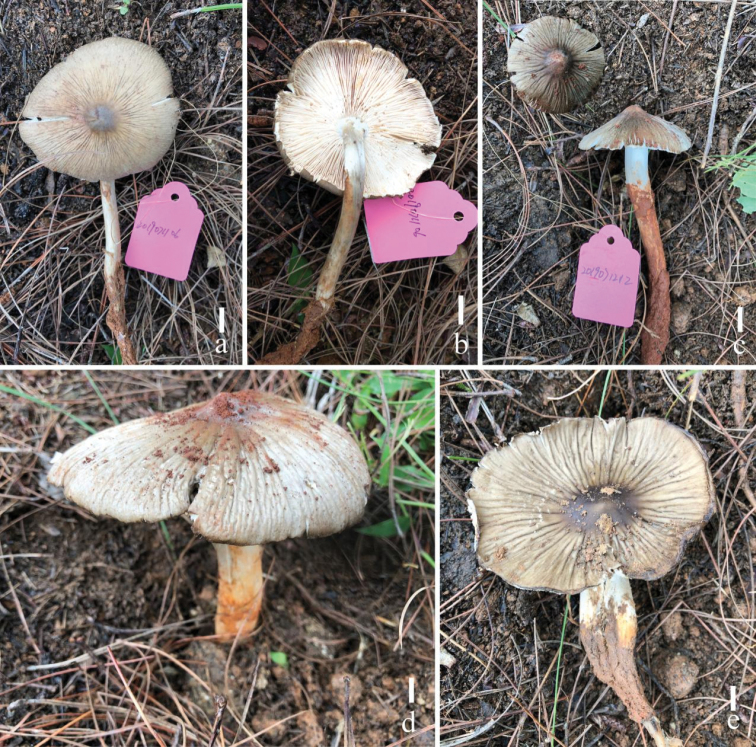
Fresh basidiomata representative of *Termitomycesyunnanensis***a, b** HKAS 124501 **c** HKAS 124502 **d** HKAS 124503 **e** HKAS 124517. Scale bars: 1 cm. Photographs by Song-Ming Tang.

**Figure 6.**
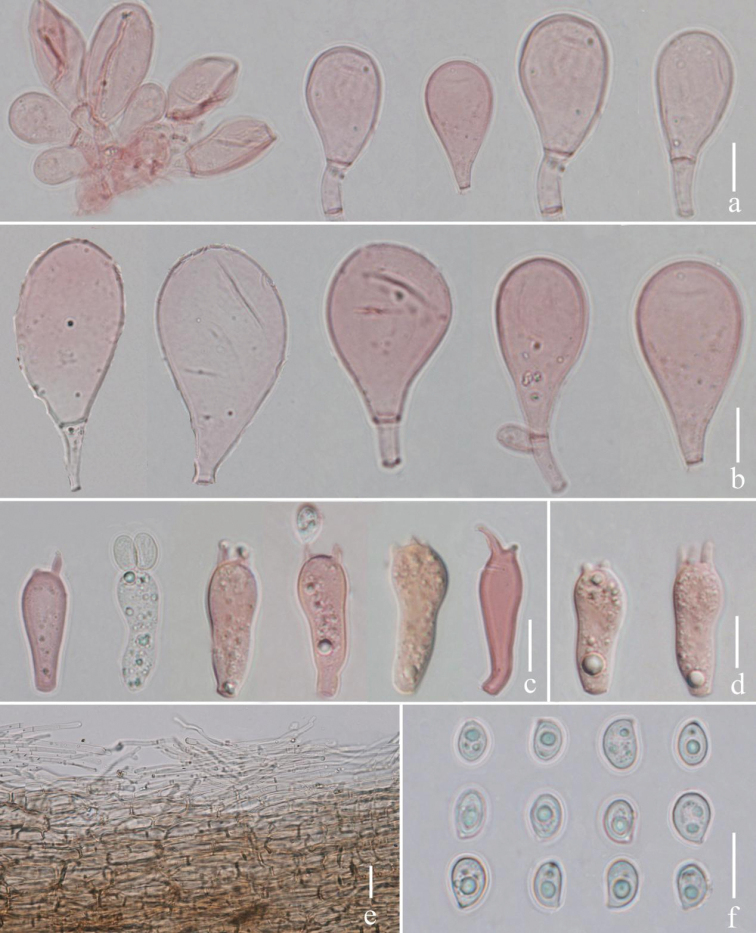
*Termitomycesyunnanensis***a** lamellar edge and cheilocystidia **b** pleurocystidia **c** basidia with acute sterigmata apex **d** basidia with obtuse sterigmata apex **e** pileipellis **f** basidiospores. Scale bars: 10 μm (**a–d, f**); 20 μm (**e**). Photographs by Song-Ming Tang.

##### ﻿Key to species of *Termitomyces* reported from China

To date, 14 *Termitomyces* species have been reported from China. However, the identification of some species, namely *T.aurantiacus*, *T.eurrhizus*, *T.entolomoides*, *T.globulus*, *T.mammiformis* and *T.tylerianus*, was based on morphology only. Further studies using DNA sequence analyses are required to confirm or inform the presence of those species in China.

**Table:** 

1	Basidiomata small, with pileus diam. ≤ 4.5 cm when mature	**2**
–	Basidiomata medium to large, with pileus diam. > 4.5 cm when mature	**5**
2	Pileus surface cream to whitish; pileus diam. 2.5–3.0 cm; perforatorium pointed, pseudorhiza long and slender, cheilocystidia and pleurocystidia absent, annulus present	***T.tylerianus***
–	Pileus surface brownish-gray, dirty white, grayish brown	**3**
3	Pseudorhiza absent or present; pileus small, diam. 1.2–2.5 cm, dirty-white, soon split at the margin	***T.microcarpus***
–	Pseudorhiza present, pileus larger	**4**
4	Pileus 2.0–4.5 cm diam., stipe white to cream, cylindrical, smooth	***T.fragilis***
–	Pileus 3.5–4.0 cm diam.; stipe pale grey, tapering upwards, floccules	***T.entolomoides***
5	Pileus white or greyish white	**6**
–	Pileus ochraceous-orange or yellowish-brown, grey to dark brown or dirty white	**7**
6	Stipe surface smooth, perforatorium obtuse, gray to brownish gray	***T.heimii***
–	Stipe surface squamulose, perforatorium mammiform, pale brown to dark brown	***T.mammiformis***
7	Pileus ochraceous-orange or yellowish-brown	**8**
–	Pileus grey to dark brown or dirty white	**9**
8	Perforatorium mucronate, pileus reddish-brown, 5–8 cm diam.; stipe white to whitish, cylindrical	***T.aurantiacus***
–	Perforatorium non-differentiated, pileus reddish-brown to yellowish-brown, 15–20 cm diam., stipe white, smooth and tapering upwards	***T.globulus***
9	Annulus present; perforatorium strongly differentiated; stipe cylindrical, pseudorrhiza black and long	***T.eurrhizus***
–	Annulus absent, pseudorrhiza white to pale yellow	**10**
10	Stipe cylindrical	**11**
–	Stipe tapering upwards	**12**
11	Pileus densely tomentose to tomentose-squamulose, regular greyish white and grey dark rimose-squamulose in dry condition	***T.intermedius***
–	Pileus surface conspicuously venose, smooth	***T.yunnanensis***
12	Stipe surface with white to yellowish-brown floccules and tapering upwards, pileus 5–22 cm diam.; perforatorium broadly round or blunt	***T.bulborhizus***
–	Stipe surface smooth	**13**
13	Stipe grey, cheilocystidia few, broadly clavate, perforatorium acute, pileus dark grey, greyish white to grey, stipe greyish white to grey on the surface	***T.tigrinus***
–	Stipe white, cheilocystidia common mostly Y-shaped, perforatorium obtuse, pileus white to cream, stipe white on the surface	***T.upsilocystidiatus***

## ﻿Discussion

In this study, we combined sequences of three non-translated loci (nrLSU, mrSSU and ITS) to carry out phylogenetic analyses of *Termitomyces* species in order to investigate the phylogenetic relationships between the two new species we described and other *Termitomyces* species.

Most *Termitomyces* species have uniform morphology, although some show extensive variability. In this study, five *T.intermedius* specimens were collected from Yunnan Province, China and showed differences in stipe surface colour and cheilocystidia shape when compared to the holotype of *T.intermedius* from Japan (Terashima et al. 2016). However, the latter, and our collections had identical DNA sequences (see above notes), which indicates their conspecificity. *Termitomycesle-testui* (Pat.) R. Heim, *T.microcarpus* (Berk. & Broome) R. Heim, *T.striatus*, and *T.schimperi* were also reported to be morphologically variable, with multiple formae described (See Index Fungorum). However, some specimens identified as *T.striatus* (DM280, DM151, BR5020212704478V, BR5020168468769 and BR5020169404421), despite showing similar morphology, clustered in different species-level clades in our phylogeny. Because of this morphological variability in some *Termitomyces* species, species identification or delineation should not be based only on morphology. Molecular analyses are also necessary to resolve the relationship between *Termitomyces* species.

In China, *Termitomyces* species are considered as delicacies, widely collected and consumed by local people, usually stir-fried with chili, bacon and garlic. They are called “Jizongjun” in Chinese, which means the taste of chicken. *Termitomyces* species are considered nutritious (a good source of proteins, lipids, crude fibres and minerals) for a daily healthy diet (Kansci et al. 2003). *Termitomyces* are an important source of income for people from rural areas of China. *Termitomycestigrinus*, *T.intermedius* and *T.yunnanensis* are commonly found in mushroom markets from July to September and often sold around 120–200 RMB/kg.

To date, 14 *Termitomyces* species have been reported in China (including the result in this study) namely *T.aurantiacus* (Yunnan and Gui zhou), *T.bulborhizus* (Sichuan and Yunnan), *T.eurrhizus* (Berk.) R. Heim (Yunnan, Henan, Guizhou, Tibet, Guangdong and Hainan), *T.entolomoides* R. Heim (Guangdong), *T.fragilis* L. Ye, Karun, J. C. Xu, K. D. Hyde & Mortimer (Yunnan), *T.globulus* R. Heim & Gooss.-Font. (Sichuan and Yunnan), *T.heimii* (Yunnan), *T.intermedius* Har. Takah. & Taneyama (Henan, Guangdong and Yunnan), *T.mammiformis* (Yunnan and Tibet), *T.microcarpus* (Yunnan, Sichuan and Guizhou), *T.tigrinus* (Yunnan), *T.tylerianus* Otieno (Yunnan and Guangdong), *T.upsilocystidiatus* (Yunnan), *T.yunnanensis* (Yunnan) (Wei et al. 2004; Huang et al. 2017; Ye et al. 2019; Tang et al. 2020). These species are mainly distributed in southern part of China.

*Termitomycestigrinus* and *T.yunnanensis* are widely distributed in the subtropical broad-leaved forests of Dali, Yuxi, Baoshan, and Chuxiong in Yunnan, where the annual average temperature is 12–22 °C, and the elevation is between 1,000–3,500 m (Xiwen and Walker 1986). *Termitomyces* species form symbiotic relationships with termites in the subfamily Macrotermitinae, and their distribution thus depends on the presence of termites. In China, Yunnan, Guangxi and Hainan provinces have a tropical to subtropical climate suitable for termites, hence the abundance of *Termitomyces* species in those provinces.

## Supplementary Material

XML Treatment for Termitomyces intermedius

XML Treatment for Termitomyces tigrinus

XML Treatment for Termitomyces yunnanensis
